# Comparison of VSV Pseudovirus and Focus Reduction Neutralization Assays for Measurement of Anti-*Andes orthohantavirus* Neutralizing Antibodies in Patient Samples

**DOI:** 10.3389/fcimb.2020.00444

**Published:** 2020-09-17

**Authors:** Cecilia Vial, Annalis Whitaker, Jan Wilhelm, Jimena Ovalle, Ruth Perez, Francisca Valdivieso, Marcela Ferres, Constanza Martinez-Valdebenito, Philip Eisenhauer, Gregory J. Mertz, Jay W. Hooper, Jason W. Botten, Pablo A. Vial

**Affiliations:** ^1^Facultad de Medicina Clínica Alemana Universidad del Desarrollo, Programa Hantavirus, Instituto de Ciencias e Innovación en Medicina, Santiago, Chile; ^2^Division of Immunobiology, Department of Medicine, University of Vermont, Burlington, VT, United States; ^3^Cellular, Molecular and Biomedical Sciences Graduate Program, University of Vermont, Burlington, VT, United States; ^4^Clínica Alemana de Santiago, Santiago, Chile; ^5^Laboratorio de Infectología y Virología Molecular, Departamento de Enfermedades Infecciosas e Inmunología Pediátrica, Facultad de Medicina Pontificia Universidad Católica de Chile, Santiago, Chile; ^6^Division of Infectious Diseases, Department of Internal Medicine University of New Mexico, Albuquerque, NM, United States; ^7^Molecular Virology Branch, United States Army Medical Research Institute of Infectious Diseases, Frederick, MD, United States; ^8^Department of Microbiology and Molecular Genetics, University of Vermont, Burlington, VT, United States

**Keywords:** *Andes orthohantavirus* (ANDV), hantavirus cardiopulmonary syndrome (HCPS), *orthohantavirus*, vesicular stomatitis virus (VSV) pseudovirus, neutralizing antibodies

## Abstract

*Andes orthohantavirus* (ANDV) is the etiologic agent of hantavirus cardiopulmonary syndrome (HCPS), which has a case fatality rate around 35%, with no effective treatment or vaccine available. ANDV neutralizing antibody (NAb) measurements are important for the evaluation of the immune response following infection, vaccination, or passive administration of investigational monoclonal or polyclonal antibodies. The standard assay for NAb measurement is a focus reduction neutralization test (FRNT) featuring live ANDV and must be completed under biosafety level (BSL)-3 conditions. In this study, we compared neutralization assays featuring infectious ANDV or vesicular stomatitis virus (VSV) pseudovirions decorated with ANDV glycoproteins for their ability to measure anti-ANDV NAbs from patient samples. Our studies demonstrate that VSV pseudovirions effectively measure NAb from clinical samples and have greater sensitivity compared to FRNT with live ANDV. Importantly, the pseudovirus assay requires less labor and sample materials and can be conducted at BSL-2.

## Introduction

*Andes orthohantavirus* (ANDV), the etiologic agent of hantavirus cardiopulmonary syndrome (HCPS) in Chile and Argentina, has a case fatality rate of 35% (Centers for Disease Control Prevention (CDC), 1997; Figueiredo et al., 2014). ANDV is the only hantavirus known to be transmissible from person to person, usually among close household contacts (Padula et al., 1998; Ferres et al., 2007). HCPS has an incubation period ranging from 10 to 49 days (Vial et al., 2006) followed by a febrile prodrome including fever, myalgia, and headache (Mertz et al., 2006). Patients can then rapidly progress to a cardiopulmonary phase, which can end in cardiogenic shock, respiratory failure, and death (Duchin et al., 1994).

Treatment for HCPS is based on critical care support, including extracorporeal membrane oxygenation in severe disease, as there are currently no viral-specific drugs or treatments available (Wernly et al., 2011). Recently, our group used passive immunotherapy to treat acutely ill HCPS patients in Chile (Vial et al., 2015). Neutralizing antibody (NAb) activity in convalescent plasma was measured by a focus reduction neutralization test (FRNT), and a dose of 5,000 U/kg was administered. Although not a controlled trial, the case fatality rate was 14% in treated patients vs. 33% in concurrent untreated patients.

The FRNT assay, which features live infectious ANDV, is the standard method to quantify hantavirus NAbs. While effective, this assay can take up to 12 days to complete and must be performed by trained laboratory personnel in a biosafety level-3 laboratory (Bharadwaj et al., 2000). Alternative methods featuring recombinant vesicular stomatitis viruses (VSV) decorated with the envelope glycoprotein of ANDV (in place of the native VSV glycoprotein) can be done at biosafety level-2 within 2 days. Importantly, these pseudovirion neutralization assays (PsVNAs) have been shown to be reproducible and sensitive (Ray et al., 2010; Brown et al., 2011; Higa et al., 2012; Li et al., 2018).

The measurement of hantavirus NAbs is important in clinical settings to measure and standardize the amount of NAbs from convalescent patient plasma given as treatment. In addition, as ANDV vaccine development progresses, quantifying levels of NAbs induced by vaccination will be a necessary readout for immunogenicity and defining correlates of protection. The goal of the current study was to evaluate and compare, in multiple laboratories, the ability of two ANDV PsVNA assays (replication-competent or -incompetent platforms) and the FRNT assay to measure NAbs in samples from ANDV patients.

## Materials and Methods

### Patients

We analyzed blood samples from 28 anonymized patients who had been previously enrolled in different protocols from our Hantavirus Program between 2001 and 2018: household contacts follow-up (Ferres et al., 2007); immune plasma treatment protocol (Vial et al., 2015); and an immune response protocol (Fondecyt 1161447 project). Stored plasma samples were obtained from whole blood drawn at the time of enrollment in the pertinent study. Peripheral blood was obtained by venipuncture, collected in ethylenediaminetetraacetic acid (EDTA) or acid citrate dextrose (ACD) blood tubes (4 ml) and separated by centrifugation at low speed (1,000 rpm for 15 min). After centrifugation, plasma was stored at −80°C until further use. ANDV-positive patients were diagnosed by the detection of anti-ANDV antibodies by ELISA or detection of the viral genome by RT-qPCR (Vial et al., 2016). We also included samples from 40 negative household contacts of HCPS cases that were obtained between 1 and 5 weeks after HCPS index case detection. These contacts were followed for 40 days, remaining serologically negative to ANDV and no symptom development.

### Cells

Human embryonic kidney (HEK 293T) cells, purchased from American Tissue Culture Collection (Manassas, VA, USA), were maintained in Dulbecco's modified Eagle medium (DMEM) (11965–092) supplemented with 10% fetal bovine serum (FBS) (16140–071), 1% penicillin-streptomycin (15140–122), 1% MEM non-essential amino acids solution (11140–050), 1% 4-(2-hydroxyethyl)-1-piperazineethanesulfonic acid (HEPES) buffer solution (15630–130), and 1% Gluta-MAX (35050–061) purchased from Thermo Fisher Scientific (Carlsbad, CA). African green monkey kidney cells (Vero E6) (kindly provided by J. L. Whitton) were grown in DMEM supplemented with 10% FBS, 1% penicillin-streptomycin, and 1% HEPES buffer solution. Cells were grown in a humidified incubator at 37°C with 5% CO_2_.

### Viruses

A single-round infectious VSV Renilla luciferase reporter virus decorated with either the native VSV glycoprotein (G) (VSVΔG/VSVG) or the ANDV glycoprotein (GP) (VSVΔG/ANDVGP) was used for PsVNA assays. These pseudovirion particles were generated as described (Higa et al., 2012). Briefly, recombinant VSV derived from a full-length cDNA clone of VSV Indiana serotype in which the VSV glycoprotein (G) gene was replaced with the Renilla luciferase (rLuc) gene (VSVΔG^*^rLuc) was used to infect HEK 293T cells previously transfected with a plasmid encoding the glycoprotein of either VSV or ANDV strain CHI-9717869. VSVΔG^*^rLuc and the VSV G-expressing plasmid were kindly donated by R. Doms, and the plasmid expressing full-length ANDV GP was provided by J. W. Hooper. The resulting VSV G- or ANDV GP-decorated pseudovirion particles are able to enter cells and express luciferase but are not able to generate new infectious particles.

Replication-competent VSV pseudovirion particles expressing the ANDV GP (CHI-9717869) in place of the native VSV G protein (rVSV/ANDVGP) were used for plaque reduction neutralization tests (PRNTs). The rVSV/ANDVGP virions were developed by Brown et al. (2011) and kindly provided by H. Feldmann.

Authentic infectious ANDV strain CHI-9717869 (provided by Dr. Jay Hooper) was used for FRNTs. Infectious stocks of this virus were grown on Vero E6 cells in the UVM BSL-3 laboratory.

### Pseudovirion Neutralization Assay

Vero E6 cells were seeded at 10,000–30,000 cells/well in a 96-well plate and grown overnight. The pseudovirions (VSVΔG/VSVG and VSVΔG/ANDVGP) were preincubated with serial dilutions of patient sera ranging from 1:50 to 1:51,200 for 30 min at 37°C. Then, 50 μl of each condition was added to cells in 96-well plates and incubated at 37°C for 1 h. Following viral absorption, fresh medium was added to the single-round infectious VSV Renilla luciferase reporter viruses (VSVΔG/VSVG and VSVΔG/ANDVGP), and 1 day later, cells were analyzed for the expression of Renilla luciferase (Ray et al., 2010; Higa et al., 2012; Kwilas et al., 2014) using the Renilla Luciferase Assay System (Promega, USA) in a luminometer (BioTek, USA). Controls in these assays included incubation of viral particles with human serum from hantavirus-negative individuals, species matched non-immune immunoglobulin G (IgG), or no antibody. Specificity was further determined through the use of VSV particles decorated with VSV G.

### Focus Reduction Neutralization Test

The FRNT was conducted in the UVM BSL-3 facility under an approved institutional biosafety protocol. Patient serum or a human polyclonal IgG control was heated for 30 min at 56°C and then diluted serially 3-fold (1:50–1:24,300) in 50 μl of complete Vero E6 cell culture media (cDMEM). Diluted patient serum was mixed with an equal volume of cDMEM containing the equivalent of ~100 focus forming units (FFUs) of ANDV and incubated for 1 h at 37°C. Media from confluent Vero E6 cell monolayers in 48-well tissue culture plates was removed, and 100 μl of the antibody–virus mixture was inoculated onto the cells and incubated at 37°C in a 5% CO_2_ incubator for 1 h, after which the wells were overlaid with 1.2% methylcellulose in cDMEM, incubated at 37°C in a 5% CO_2_ incubator for 12 days, and then fixed in 25% formaldehyde in 3 × phosphate buffered saline (PBS). Cells were permeabilized with 0.1% 100X Triton in 1 × PBS for 15 min and then incubated with the primary rabbit anti-ANDV N polyclonal antibody (NR-9673; BEI Resources) (1:20,000) followed by a peroxidase-labeled goat anti-rabbit antibody (5220-0336; SeraCare) (1:2,000) and then the peroxidase substrate (5510-0030; SeraCare). Images of the wells were captured using an Alpha Innotech imager, and viral foci were quantified manually.

### Plaque Reduction Neutralization Assay

For the replication-competent rVSV/ANDVGP virus, a plaque reduction neutralization assay (PRNT) was used. Patient serum was heated for 30 min at 56°C and diluted in the same range as described for the FRNT method. Diluted patient serum or a human polyclonal IgG control was mixed with an equal volume of cDMEM containing ~100 plaque-forming units (PFUs) of rVSV/ANDVGP and incubated for 1 h at 37°C. Medium from confluent Vero E6 cell monolayers in 24-well tissue culture plates was removed, and 100 μl of the antibody–virus mixture was inoculated onto the cells and incubated at 37°C in a 5% CO_2_ incubator for 1 h, after which the wells were overlaid with 1.4% agarose in cDMEM and incubated at 37°C in a 5% CO_2_ incubator for 2 days. Viral plaques from areas of cell death were visualized following fixation and staining with crystal violet (Brown et al., 2011).

### Statistical Analysis

Neutralizing antibody titers were defined as the reciprocal of the highest serum dilution that resulted in an 80% reduction in the number of viral foci (FRNT_80_) or plaques (PRNT_80_) compared to virus controls in duplicate assays or reciprocal of the highest serum dilution that resulted in an 80% reduction of luciferase measurement in the PsVNA. We performed a descriptive analysis using frequency distribution and percentages. To compare different test performances, we used Spearman correlation test in RStudio v1.1.463.

## Results

**Figure 1:**
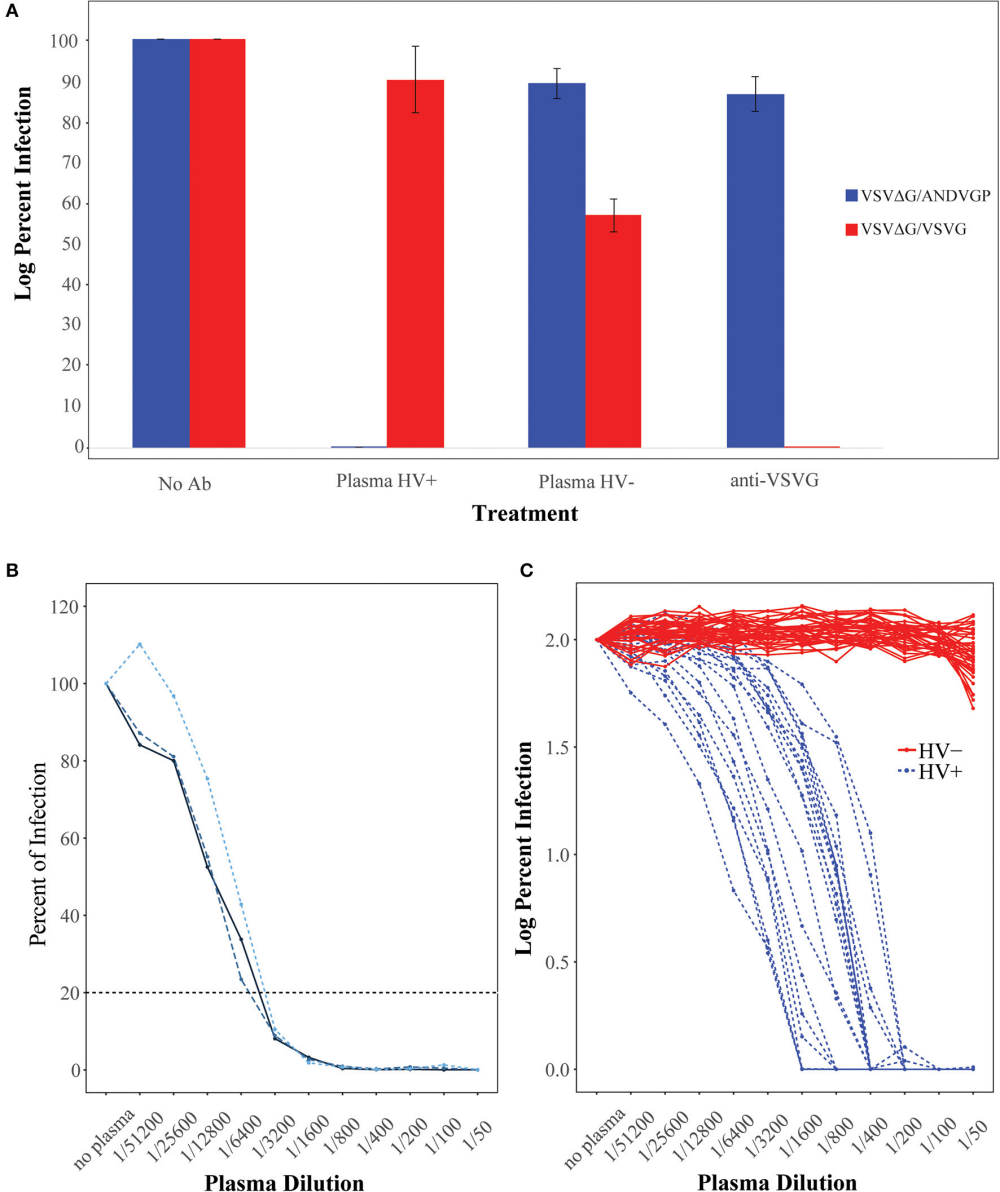
Characterization of *Andes orthohantavirus* (ANDV) glycoprotein (GP)- or vesicular stomatitis virus (VSV) G-decorated Renilla luciferase reporter viruses. **(A)** VSVΔG/ANDVGP (blue) and VSVΔG/VSVG (red) pseudovirus (Psdv) infectivity in Vero E6 cells. Psdv infection (percent of infection) was measured with (i) medium alone (no Ab), (ii) hantavirus-positive patient plasma (HV+) diluted 1/50, (iii) hantavirus-negative patient plasma (HV–) diluted 1/50, and (iv) an anti-VSV G antibody diluted 1/50. Each bar represents the mean and standard error. **(B)** Three independent experiments of full titration curves for one plasma sample from a hantavirus-positive patient. Percent of infection by VSVΔG/ANDVGP was measured by luciferase activity (RLU) at each plasma dilution and neutralizing antibody (NAb) titer calculated when there was an 80% inhibition of virus infection. **(C)** Full titration curves of 60 human plasma samples. Sera from 20 hantavirus-positive donors (HV+ in blue) and 40 hantavirus-negative donors (HV– in red) were tested for NAbs against VSVΔG/ANDVGP. Y axis shows the percent of infection (log_10_) of each dilution point.

We initially used the single-round infectious VSVΔG/ANDVGP reporter viruses to measure NAb titer in 20 hantavirus-positive (Plasma HV+) and 40 hantavirus-negative plasma samples (Plasma HV–) (Figure 1). Figure 1A shows the infection percentage measured when no antibody is present, and it demonstrates the specificity of the ANDV GP-decorated VSV luciferase virus (VSVΔG/ANDVGP) for the measurement of anti-ANDV NAbs. Both VSVΔG/ANDVGP and VSVΔG/VSVG readily infected cells when no antibody was added and served in each replicate experiment as a control. The VSV-specific NAb I14 effectively neutralized VSVΔG/VSVG but not VSVΔG/ANDVGP; likewise, specific inhibition is shown when hantavirus-positive serum samples are used, blocking infectivity of VSVΔG/ANDVGP but not VSVΔG/VSVGP. Furthermore, Figure 1B illustrates the reproducibility of results obtained in one plasma sample tested in three independent experiments each in triplicate, giving an 80% inhibition titer of ~1:3,200 in each replicate. Finally, when this assay was used on our panel of 20 hantavirus-positive plasma samples and 40 hantavirus-negative samples, there was clear specificity (Figure 1C), as only plasma from hantavirus-positive donors could neutralize the ANDV GP-decorated pseudoparticles (VSVΔG/ANDVGP). NAb titers measured for these samples are shown in Table 1, including plasma donation date measured as time after infection. Notably, our results demonstrate that hantavirus patients retained considerable levels of NAbs for at least 10 years following infection.

**Table 1:** Titer of NAb in plasma of ANDV patients as measured using the ANDV GP-decorated Renilla luciferase reporter virus (VSVΔG/ANDVGP).

**Plasma donor**	**Age at infection**	**Gender**	**Interval following ANDV infection to plasma donation (months)**	**PsVNA80 NAb titer**
1	18	Male	60	6,400
2	41	Male	50	800
3	49	Male	16	1,600
4	47	Male	9	3,200
5	27	Male	87	800
6	49	Female	82	800
7	14	Female	124	6,400
8	28	Male	68	800
9	33	Female	108	800
10	21	Female	16	3,200
11	29	Male	121	800
12	20	Female	30	3,200
13	42	Male	41	400
14	39	Male	45	3,200
15	24	Female	24	800
16	17	Female	28	6,400
17	14	Male	83	3,200
18	42	Male	22	1,600
19	28	Male	22	800
20	33	Male	15	6,400

*ANDV, Andes orthohantavirus; GP, glycoprotein; Nab, neutralizing antibody; PsVNA, pseudovirion neutralization assay*.

**Figure 2:**
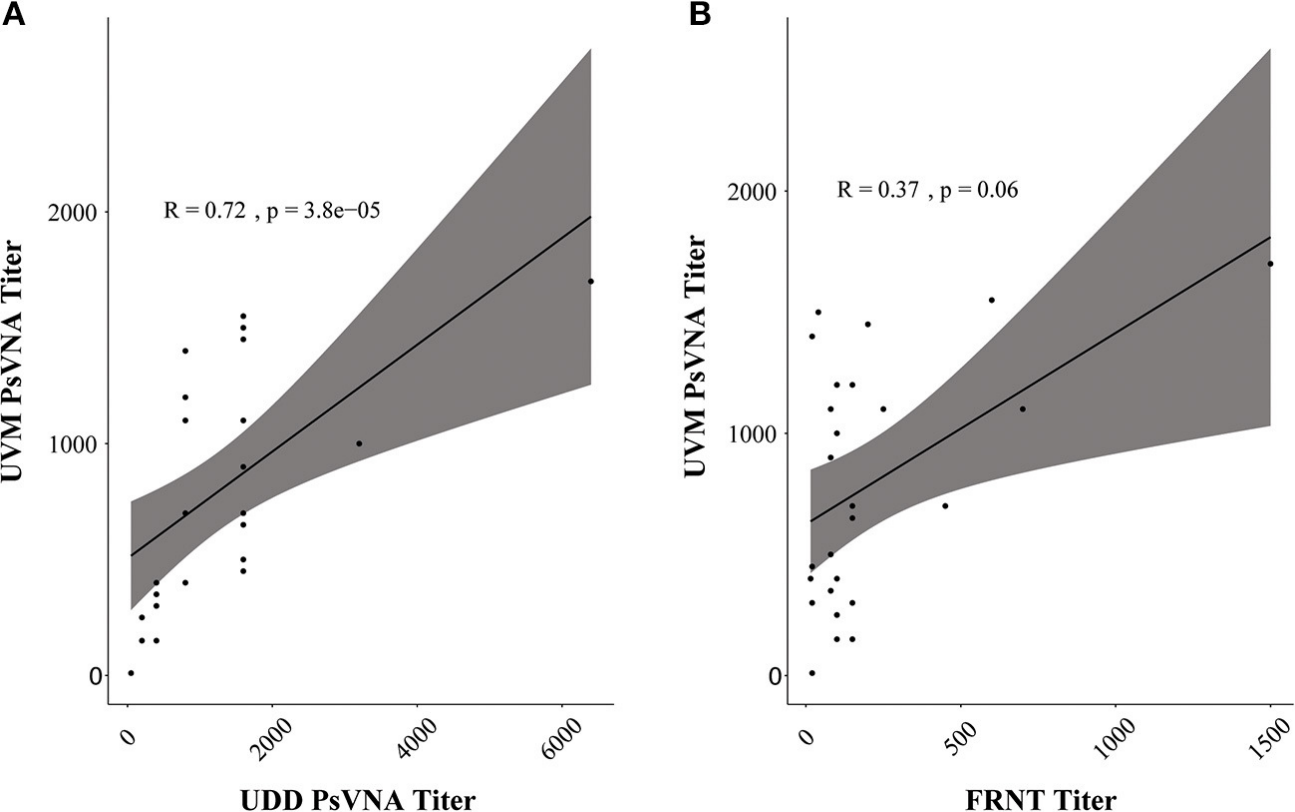
Pseudovirion neutralization assay (PsVNA) titer measurement correlation. **(A)** Spearman correlation of neutralizing antibody (NAb) titer measured by vesicular stomatitis virus (VSV)ΔG/*Andes orthohantavirus* (ANDV) glycoprotein (GP) luciferase assay (PsVNA) performed in Chile [Universidad del Desarrollo (UDD)] vs. replication-competent rVSV/ANDVGP plaque reduction neutralization test (PRNT) assay carried out in Vermont [University of Vermont (UVM)]. In gray is the confidence interval (95%). **(B)** Spearman correlation of NAb titer measured at UVM *via* replication-competent rVSV/ANDVGP PRNT vs. orthohantavirus focus reduction neutralization test (FRNT). In gray is the confidence interval (95%).

We next compared the performance of replication-incompetent VSVΔG/ANDVGP (PsVNA) to that of replication-competent rVSV/ANDVGP (PRNT), as well as to authentic infectious ANDV (FRNT). Twenty-six samples from eight HCPS patients at progressive time points after hospital admission were screened for NAbs. The single-round infectious VSVΔG/ANDVGP PsVNA was tested at the Universidad del Desarrollo in Chile, while the replication-competent rVSV/ANDVGP PRNT and infectious ANDV FRNT were conducted at the University of Vermont in the USA. Table 2 shows the NAb titers obtained by each center/technique. Using the Spearman correlation coefficient to compare each test result (Figure 2), we found a stronger correlation between the results obtained using the replication-competent (PRNT) and -incompetent (PsVNA) assays at different sites (*R* = 0.76, *p* = 3.802e−05) than comparing results obtained from the replication-competent rVSV/ANDVGP PRNT vs. standard FRNT at the same site. One consistent trend was that NAb titer was lower when measured by FRNT vs. either PsVNA or PRNT assay. Importantly, both VSV pseudovirus assays appear to measure the same kinetic trends (Table 2) captured *via* FRNT, further illustrating their relevance for measuring NAbs in clinical samples. Notably, our results demonstrate a continuous increase in NAb titers even 60 days after hospital admission of patients.

**Table 2:** Kinetics of NAb formation in ANDV patients as measured by standard FRNT or ANDV GP-decorated VSV pseudoviruses (single-round infectious VSVΔG/ANDVGP luciferase reporter virus or replication-competent rVSV/ANDVGP virus) at two different centers: UDD and UVM.

**Patient number**	**Day post-hospitalization**	**UDD_replication-incompetent VSVΔG/ANDVGP luciferase**	**UVM_replication-competent VSV/ANDVGP**	**UVM_FRNT**
21	1	200	150	150
	4	1,600	500	80
	6	1,600	1,500	40
	70	6,400	1,700	1,500
22	2	400	350	80
	4	1,600	450	20
	6	1,600	900	80
	62	1,600	700	450
23	2	400	400	100
	4	800	1,100	80
	6	800	400	15
	66	1,600	650	150
24	1	400	300	150
	4	400	300	20
	5	800	700	150
	56	1,600	1,100	700
25	6	800	1,200	100
	8	800	1,400	20
	10	800	1,200	150
	79	1,600	1,550	600
26	1	50	–	20
	3	200	250	100
	6	800	1,100	250
	63	3,200	1,000	100
27	1	1,600	1,450	200
28	1	400	150	100

*ANDV, Andes orthohantavirus; FRNT, focus reduction neutralization test; GP, glycoprotein; NAb, neutralizing antibody; UDD, Universidad del Desarrollo; UVM, University of Vermont; VSV, vesicular stomatitis virus*.

## Discussion

Currently, hantavirus neutralization assays are relatively time-consuming and require the use of viruses in high-containment (BSL-3) facilities. Several studies have demonstrated that hantavirus glycoproteins can be pseudotyped onto VSV (Ogino et al., 2003; Ray et al., 2010; Higa et al., 2012) and lentiviral vectors (Cifuentes-Muñoz et al., 2010). Such systems present a safe and effective method for studying virus entry, as they are produced at reasonable titers and require only BSL-2 conditions. In this study, we compared three assays (replication-competent VSV PRNT, replication-incompetent VSV PsVNA, or FRNT with infectious ANDV) for their ability to measure anti-ANDV NAbs in clinical samples.

VSV pseudovirions decorated with ANDV GP can effectively and reproducibly measure NAbs from ANDV patient sera and appear to have higher sensitivity when compared to FRNT featuring authentic ANDV. Because of the reduced cost, biosafety requirements, and time to complete, the pseudovirus assays may offer a practical advantage over standard FRNT.

Our examination of ANDV patients resulted in two general findings. First, NAbs steadily increase over the course of acute infection up to 60 days post-infection. Second, the anti-ANDV NAb is extremely durable, lasting at least 10 years after infection and suggest that long-term protective immunity is possible. Further, it also indicates individuals can remain robust sources of convalescent plasma for long periods. Based on our finding of low NAb titers early during acute infection and the apparent efficacy of immune plasma treatment in our previous study (Vial et al., 2015), we suggest that administering convalescent serum in the early stages of HCPS could be helpful in fighting this disease by augmenting NAb titers.

The Chilean Department of Health is implementing an HCPS survivors plasma bank to treat acute HCPS patients. The assays we describe could be used to identify donors with clinically relevant levels of NAb. Further, they could provide a platform for the evaluation of NAb induction by candidate ANDV vaccines and to further studies on the natural history of the B cell response to ANDV infection.

## Data Availability Statement

The raw data supporting the conclusions of this article will be made available by the authors, without undue reservation.

## Ethics Statement

The studies involving human participants were reviewed and approved by Ethics committee at Clínica Alemana Universidad del Desarrollo IRB4858. Written informed consent to participate in this study was provided by the participant or participant's legal guardian/next of kin.

## Author Contributions

CV, JW, JO, PV, JB, MF, CM-V, and FV contributed to the conceptualization. FV, PV, JW, JH, CV, AW, and JB contributed to the methodology. JO and AW contributed to the formal analysis. CV, JB, PV, and GM contributed to writing the original draft preparation. JH, MF, CM-V, and AW contributed to writing, reviewing, and editing. RP and PE contributed to project administration. GM, CV, MF, PV, and JB contributed to funding acquisition. All authors have read and agreed to the published version of the manuscript.

## Conflict of Interest

The authors declare that the research was conducted in the absence of any commercial or financial relationships that could be construed as a potential conflict of interest. The handling editor declared a past co-authorship with one of the authors JH.
